# DNA Nanostructures for Targeted Antimicrobial Delivery

**DOI:** 10.1002/anie.202002740

**Published:** 2020-05-20

**Authors:** Ioanna Mela, Pedro P. Vallejo‐Ramirez, Stanislaw Makarchuk, Graham Christie, David Bailey, Robert M. Henderson, Hiroshi Sugiyama, Masayuki Endo, Clemens F. Kaminski

**Affiliations:** ^1^ Department of Chemical Engineering and Biotechnology University of Cambridge Philippa Fawcett Drive Cambridge CB3 0AS UK; ^2^ Department of Pharmacology University of Cambridge Tennis Court Road Cambridge CB2 1PD UK; ^3^ Department of Chemistry Graduate School of Science Kyoto University Kitashirakawa-oiwakecho, Sakyo-ku Kyoto 606-8502 Japan; ^4^ Institute for Integrated Cell Material Sciences Kyoto University Yoshida-ushinomiyacho, Sakyo-ku Kyoto 606-8501 Japan

**Keywords:** atomic force microscopy, antimicrobial, bionanotechnology, DNA nanostructures, *d*STORM

## Abstract

We report the use of DNA origami nanostructures, functionalized with aptamers, as a vehicle for delivering the antibacterial enzyme lysozyme in a specific and efficient manner. We test the system against Gram‐positive (*Bacillus subtilis*) and Gram‐negative (*Escherichia coli*) targets. We use direct stochastic optical reconstruction microscopy (*d*STORM) and atomic force microscopy (AFM) to characterize the DNA origami nanostructures and structured illumination microscopy (SIM) to assess the binding of the origami to the bacteria. We show that treatment with lysozyme‐functionalized origami slows bacterial growth more effectively than treatment with free lysozyme. Our study introduces DNA origami as a tool in the fight against antibiotic resistance, and our results demonstrate the specificity and efficiency of the nanostructure as a drug delivery vehicle.

## Graphical abstract

**Antibiotic resistance** is a growing health issue that is now rendering humans vulnerable once again to infections that have been treatable for decades. Various approaches have been proposed to overcome this threat and effectively treat bacterial infections. DNA nanostructures, functionalized with aptamers, were used as a vehicle for delivering the antibacterial enzyme lysozyme in a specific and efficient manner, to destroy bacterial targets.

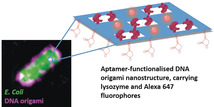

Antibiotic resistance is a growing worldwide human health issue, and alternative antimicrobial strategies are needed urgently. Several novel materials, including metal–organic frameworks,^1^ antimicrobial peptides,^2^ nanoparticles^3^ and combinations of these,^4^ have shown promise for new antimicrobial strategies, but metal‐based materials have low stability and/or can be highly toxic to mammalian cells^5^, ^6^ and methods for targeted delivery of antimicrobial peptides are lacking. In this study, we demonstrate the potential of DNA origami nanostructures functionalized with aptamers as a vehicle for delivering active antimicrobial components in a target‐specific and efficient manner. DNA origami structures are two‐ or three‐dimensional nanostructures made by exploiting the base‐pairing property of DNA.^7^ A large number (150–200) of oligonucleotides, referred to as staples, are used to fold the DNA into a pre‐designed conformation and hold it together, and these staples can be functionalized to carry various payloads.^8^, ^9^ Previous studies have shown that DNA origami has excellent biocompatibility, triggers no immune response and is reportedly stable for 12 h in vivo^10^, ^11^, ^12^, ^13^, ^14^ (and can be chemically modified to increase its stability^15^), making it an ideal candidate material for the manufacturing of highly specific drug delivery vehicles. The concept of DNA origami as a vehicle for the targeted delivery of drugs or enzymes has been explored in the context of cancer therapy and DNA nanostructures have been used to target active therapeutic molecules to eukaryotic cancer cells, resulting in the death of these cells.^10^, ^11^, ^16^, ^17^ However, there are no reports on the use of DNA origami to target bacterial cells.

In this study, we used DNA origami as a vehicle for targeted delivery of the antimicrobial enzyme lysozyme to two different bacteria in vitro. The same nanostructure was functionalized to target Gram‐positive and Gram‐negative bacteria with high specificity. We synthesized and used a previously reported DNA origami nanostructure,^18^ which consists of a frame containing five “wells” to carry molecular payloads, and functionalized it with aptamers designed to target *E. coli* and *B. subtilis* bacterial strains^19^ (Figure 1). We chose a nanostructure with “wells” as we are aiming to deliver the antimicrobial lysozyme to the surface of the bacterium. By binding the lysozyme onto a flexible linker that protrudes into the well, we maximize the chances of the active enzyme coming into contact with the bacterial surface because it can access this surface regardless of which side of the nanostructure attaches. This is important because unlike nanostructures that are targeted to mammalian cells, nanostructures targeted to bacteria will not be endocytosed, making access to the surface crucial.

**Figure 1:**
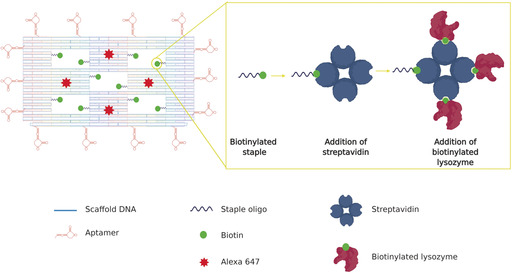
Schematic representation of the DNA origami nanostructure (left). Each of the 5 “wells” in the origami tile carries two biotinylated staples for the attachment of streptavidin and, subsequently, biotinylated lysozyme (right). Fourteen aptamers, hybridized with staples at the four sides of the DNA origami, drive the attachment of the nanostructures to the bacterial targets. Four Alexa 647 molecules act as detection beacons for the nanostructure.

Successful formation of the DNA frames was verified with atomic force microscopy (AFM). Approximately 80±8 % (*n*=5, 150 frames in each) of the frames had formed as designed. (Figure 2 a and Figure S1 in the Supporting Information). The mean measured length and width of the frames (ca. 100×100 nm; *n*=105) agreed with those of the frame design. The wells (Figure 2 a,b) measured circa 20×15 nm. The design of the frames incorporated three different functionalizations. The first was inclusion of ten biotinylated staples (two in each well, detailed sequences in the Supporting Information) to enable attachment of biotin‐tagged lysozyme to the frames. Lysozyme is an antimicrobial enzyme that breaks down peptidoglycan in the bacterial cell wall. It is effective against Gram‐positive bacteria, which have exposed cell walls, but is largely ineffective against Gram‐negative bacteria because their cell wall is protected by an outer lipid membrane. However, previous work has shown that targeted, localized delivery of lysozyme can increase its efficiency against Gram‐negative strains.^20^

**Figure 2:**
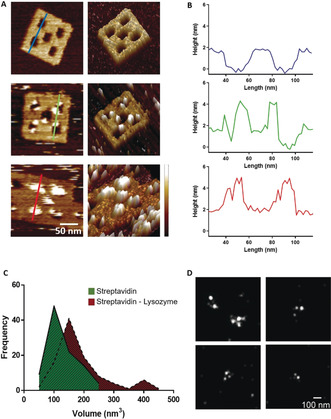
a) 2D (left) and 3D (right) atomic force microscopy images of DNA origami nanostructures before any incubation (top), after incubation with streptavidin (middle), and after successive incubations with streptavidin and biotinylated lysozyme (bottom) (height scale 0–3.5 nm, from darker to lighter). b) Cross‐sections of the nanostructures shown in (a) show the height change at the wells before any incubation (blue), after incubation with streptavidin (green) and after successive incubations with streptavidin and biotinylated lysozyme (red). c) Volume measurements of the particles bound to the DNA origami structures after incubation with streptavidin alone and streptavidin and biotinylated lysozyme. d) *d*STORM super‐resolution microscopy images of Alexa 647 fluorophores in the DNA origami nanostructures.

To attach the biotinylated lysozyme, we exploited the strong and efficient binding between biotin and streptavidin and the tetrameric structure of streptavidin that enables it to bind to four biotin molecules simultaneously. The biotin was attached to the oligonucleotide staples through a five‐base linker (ca. 2 nm in length) to provide flexibility for the streptavidin/lysozyme complex to protrude outside the well. The distance between the two biotins in the well is at least 16 nm and the theoretical diameter of a streptavidin molecule is circa 6 nm.^21^ This suggests that only one of the two biotins can bind to a streptavidin molecule at any one time. The rationale of the design is to increase the binding probability for streptavidin on the carrier. To confirm successful attachment of lysozyme to the DNA origami frames, we used AFM to measure the volumes of molecules that were bound to the frames after incubation, first with streptavidin alone and with streptavidin and biotinylated lysozyme in combination (Figure 2 c). The mean volume of bound molecules after incubation with streptavidin alone was circa 110 nm^3^ (109.3±6.3 nm^3^, *n*=150), which corresponds to the theoretical volume of streptavidin (104.5 nm^3^ for an assumed spherical protein of molecular weight 55 kDa,^21^ see Section 2.2 in Supporting Information ). The streptavidin molecules are seen on either side of the wells. The mean volume of bound molecules after incubation with streptavidin and biotinylated lysozyme was circa 160 nm^3^ (159.2±11.2 nm^3^, *n*=150), corresponding to the expected volume when 1–2 lysozyme molecules are bound to each streptavidin tetramer (theoretical volume of lysozyme is calculated at 27 nm^3^). Three or four wells per frame were occupied by streptavidin/lysozyme complexes.

The second functionalization was the inclusion of four fluorophore (Alexa 647) molecules to enable detection of the nanostructures with fluorescence microscopy. We confirmed successful incorporation of the Alexa 647‐functionalized staples with *d*STORM super‐resolution microscopy. A mean of three fluorophores were observed to be incorporated into each structure (*n*=50) (Figure 2 d). The measured distance between the single fluorophores was 62.3±17 nm (*n*=40), agreeing with the theoretical distance between the Alexa 647 molecules on the nanostructure. We observed three different populations of fluorescently labelled origami structures, with two, three, or four fluorophores attached. This could be due to low efficiency of the functionalization of the staples carrying the fluorophore, due to a molecular threading effect,^22^ in which the fluorophores on different planes miss detection by the excitation laser or due to deformed origami nanostructures.

The third functionalization was the incorporation of aptamers around the perimeter of the frame. Previous studies have shown that aptamers effectively and selectively bind to bacterial targets.^19^, ^23^ To ensure effective aptamer‐driven binding of the DNA origami to bacterial targets, we incorporated 14 aptamers in each nanostructure (Figure 1). We used aptamers that are 40 bases long and have been developed to bind to five Gram‐positive and Gram‐negative bacteria strains, including *E. coli* and *B. subtilis*, using sequential whole‐cell selection.^19^ Therefore, the exact binding sites on the bacterial surface are currently unknown. Successful binding of the DNA origami nanostructures to Gram‐positive (*B. subtilis*) and Gram‐negative (*E. coli*) bacterial strains was confirmed with structured illumination microscopy (SIM). We used *E. coli* BL21(DE3) that expresses GFP and *B. subtilis* (BS168) that was stained with Nile Red dye. Expression of GFP is visible within the whole bacterial cell, while Nile red, a lipophilic dye, stains only the outer membrane of the bacterium. As a negative control, we used *Lactococcus lactis* NZ9000 cells, which were also stained with Nile Red. Bacteria were incubated with DNA origami, and SIM was used to visualize the bacteria and the Alexa 647‐labelled DNA origami in each sample (Figure 3 a,b). The surface area of bacteria covered by DNA origami was measured by evaluating the fluorescence overlap. We observed that 83±5 % of the *E. coli* bacteria (*n*=825) had some degree of DNA origami decoration, while 72±19 % of the *B. subtilis* population (*n*=750) was decorated with DNA origami. Interestingly, in both strains, the average area of the bacterium covered by DNA origami, was circa 20 % (*n*=825 for *E. coli* and *n*=750 for *B. subtilis*, Figure 3 c). We observe a difference in the way the Gram‐negative and Gram‐positive bacteria appear to be covered by the DNA nanostructures. We do not currently know the reason for this difference in the coverage but speculate that this is influenced by two parameters, namely differences in the affinities of the nanostructures for the two bacterial strains and differences in the way the nanostructures interact with the bacteria because interactions on the surface of Gram‐negative and Gram‐positive strains are not the same. The observed coverage was achieved with a DNA origami concentration of circa 10 nm, indicating that the nanostructures have high affinity for the bacterial targets (see Supporting Information Section 5). Binding of DNA origami with no aptamers was minimal (less than 2 % area covered); the binding observed probably resulted from electrostatic interactions (Figure 3 d,e). Similarly, negligible binding was observed with use of *L. lactis*, to which aptamers cannot bind (Figure 3 f). In the *B. subtilis* sample, we noticed the presence of “minicells”, (blue arrows in Figure 3 b). Both Gram‐positive and Gram‐negative bacteria have the ability to form minicells but little is known about why this happens. A recent study^24^ suggests that minicells could act as a “damage disposal” mechanism for proteins damaged by antibiotics.

**Figure 3:**
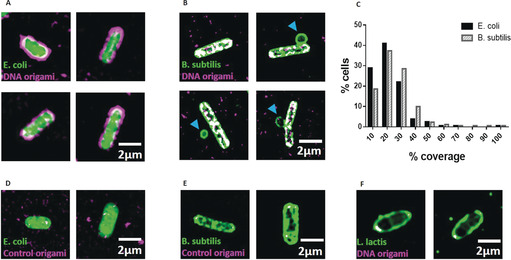
a) SIM imaging demonstrates that DNA origami binds to *E. coli*. DNA origami is magenta and GFP‐expressing *E. coli* are green, while the overlapping areas are in white. b) SIM imaging demonstrating that DNA origami binds to *B. subtilis*. DNA origami is magenta and *B. subtilis* green, with overlapping areas in white. c) The mean coverage for *E. coli* is 18.6 %, and 22.5 % for *B. subtilis*. d) *E. coli* and e) *B. subtilis* that were incubated with DNA origami that did not carry aptamers. f) SIM image of *L*. *lactis* incubated with aptamer‐functionalized DNA origami.

We used growth assays for *E. coli* and *B. subtilis* to investigate how free lysozyme, plain DNA origami, and DNA origami carrying lysozyme affected bacterial growth over 16 h (bacterial growth reached a plateau and no further growth was seen beyond 8 hours (Figure S6). To extract the growth rate in each condition, we fitted the growth curves with a modified Gompertz growth equation^25^ [Equation (1)]:

(1) $$W(t)=A\exp\left(-\exp\left(\frac{eK_{z}}{A}\left(T_{\mathrm{Lag}}-t\right)+1\right)\right)$$

Where *K*_z_ is the absolute growth rate (i.e., tangent to the curve) and *T*_Lag_ represents the time between recovery of the microbial population from being transferred to a new habitat and the occurrence of substantial cell division. In the present case, the dependent variable *W*(*t*) represents the change in OD_600_ as a function of time (*t*). The advantage of this re‐parameterization is that the growth rate coefficient (*K*_z_) constitutes the absolute growth rate at inflection, and that *A* (the upper asymptote) does not affect this parameter.^26^ Nine individual growth curves were analyzed in each condition.

The growth of the Gram‐positive bacteria (*B. subtilis*) (Figure 4 a,b) was not significantly affected by 300 nm free lysozyme but was significantly slowed by the presence of aptamer‐functionalized DNA origami carrying the same concentration of lysozyme. However, plain, aptamer‐functionalized DNA origami did not affect the growth of *B. subtilis*. In combination, these observations demonstrate that targeted delivery of the active antimicrobial enzyme increases its efficiency against bacterial targets. The growth of Gram‐negative bacteria (*E. coli*) (Figure 4 c,d) was not significantly affected by free lysozyme (300 nm), as expected.^27^, ^28^ Surprisingly, their growth was significantly slowed by the presence of plain DNA origami (aptamer‐functionalized but without any active payload), which was not the case for *B. subtilis*. Their growth was also slowed by DNA origami carrying lysozyme, but the effect beyond that of plain DNA origami was limited, indicating that targeted delivery of the enzyme in this context did not overcome the enzyme's inactivity against Gram‐negative targets. This is indicative of the inability of the enzyme to act against the Gram‐negative outer membrane, rather than inability of the nanostructures to deliver the active antibacterial. More targeted payloads can be envisaged for Gram‐negative bacteria, such as antimicrobial peptides and small molecules.

**Figure 4:**
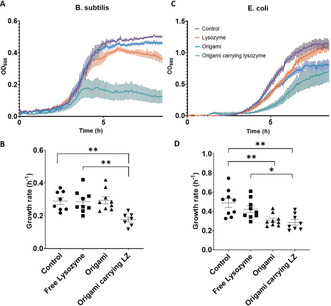
a) Averaged growth curves for *B. subtilis* (*n*=9). b) Growth rate analysis for *B. subtilis*. c) Averaged growth curves for *E. coli* (*n*=9). d) Growth rate analysis for *E. coli*. All the origami nanostructures used in those experiments were functionalized with aptamers. Error bars in each graph represent the standard error of the means.

The reduction in the growth rate observed with plain DNA origami indicates that the binding of the nanostructures to the *E. coli* interferes with their ability to divide and grow. *E. coli* grown in the presence of DNA origami that did not carry aptamers were not affected (Figure S5), indicating further that the binding of the nanostructures onto the bacterial targets slows the growth rate. This observation, together with the fact that aptamer‐functionalized DNA origami did not affect the growth of *B. subtilis*, leads us to believe that the precise nature of the interaction between the aptamer‐derivatized nanostructures and the bacterial surface directly influences the effects of the nanostructures themselves on bacterial growth.

The exact nature of this interaction is therefore an important area for future investigation and could be exploited to develop highly selective and potent antibacterial DNA nanostructures.

To ensure that the functionalized DNA origami has no detrimental effect on mammalian cells, which is a key requirement for the future development of an in vivo therapeutic strategy, we tested the effect of lysozyme carrying DNA origami on mammalian COS‐7 cells. As controls, we incubated these cells also with free lysozyme. No significant effects were observed in the viability of the cells (Figure S.7), indicating that DNA origami is a promising candidate for drug delivery in vivo.

To conclude, we have developed a platform for bacterial targeting based on the combination of DNA origami and aptamer nanotechnology. Our DNA nanostructures can bind to designated bacterial targets and deliver the antibacterial enzyme lysozyme to slow bacterial growth. Targeted and localized delivery of multiple lysozyme molecules per bacterial cell reduces the quantity of active agent required to achieve a given antibacterial effect. Our study opens the way for the use of DNA origami as a tool in the fight against antibiotic resistance, allowing for precise pathogen targeting and for the delivery of individual or combined antimicrobial compounds. The study of synergistic effects between DNA nanostructure delivery and antibiotic function is an area that can yield significant advantages in terms of reducing the dosage of antibiotic needed or the potentiation of existing antibiotic treatments. The system can be easily adapted to carry appropriate payloads for various targets, making it an attractive option for antimicrobial drug delivery. Moreover, our aptamer‐derivatized origami has the potential to target and block specific targets on the bacterial surface, thus inhibiting crucial bacterial functions. In that way, a “double‐hit” approach can be achieved, in which the bacterium is already at a disadvantage, making it easier to destroy with antimicrobial agents.

## Conflict of interest

The authors declare no conflict of interest.

## Supporting information

As a service to our authors and readers, this journal provides supporting information supplied by the authors. Such materials are peer reviewed and may be re‐organized for online delivery, but are not copy‐edited or typeset. Technical support issues arising from supporting information (other than missing files) should be addressed to the authors.

Supplementary
