# Supplementary material for: DNA Nanostructures for Targeted Antimicrobial Delivery
Source: Angew Chem Int Ed Engl. 2020 May 20;59(31):12698–702. doi: 10.1002/anie.202002740 (PMC7496991; doi:10.1002/anie.202002740)
Supplement: Supplementary file 1 — Supplementary [file ANIE-59-12698-s001.pdf]

Angewandte Chemie
Eine Zeitschrift der Gesellschaft Deutscher Chemiker

Supporting Information

DNA Nanostructures for Targeted Antimicrobial Delivery

Ioanna Mela, Pedro P. Vallejo-Ramirez, Stanislaw Makarchuk, Graham Christie, David Bailey, Robert M. Henderson, Hiroshi Sugiyama, Masayuki Endo, and Clemens F. Kaminski**

anie_202002740_sm_miscellaneous_information.pdf

Supporting information

Table of contents

Section 1. Synthesis and characterisation of DNA origami nanostructures

Section 2. Imaging of the nanostructures

Section 3. Bacterial strains imaging

Section 4. Bacterial growth curves

Section 5. Binding affinity of DNA nanostructures

Section 6. Cell viability assay

Section 7. Enzyme activity in the context of targeted delivery through DNA nanostructures

Section 1. Synthesis of DNA origami nanostructures

1.1 Synthesis of DNA origami nanostructures

We used M13mp18 single-stranded DNA as scaffold (New England Biolabs) and complementary oligonucleotides (Integrated DNA Technologies) to synthesise DNA origami nanostructures. 159 complementary oligos, as described by Yoshidome *et al*[1]. fully occupy the whole length of the scaffold. The sequences of the oligonucleotides are listed in Table S-1, in a 5′ to 3′ orientation.

Sequence Name	Sequence	Bases
5wf-001 1A-hp	TAA AAA TAC CGA ACG ACC TAA AAC TCC TCT TTT GAG GAA CAA GTT TTC TTG TAT CGC CAT TTT GCA GAT TC	71
5wf-002 1B-hp	ACC AGT CAT GGA TTA TTC CTC TTT TGA GGA ACA AGT TTT CTT GTT TAC ATT GTT TTA TTA GTA A	64
5wf-003 1C-hp	TAA CAT CAT AGC AAT ATC CTC TTT TGA GGA ACA AGT TTT CTT GTC TTC TTT GTT TTG CCA GAA T	64
5wf-004 1DE	CCT GAG AAT AGA CAG GAA CGG TAC TTT TTG CTT TGA CGA GCA CGG GGC GCG TAC TAT GGT TTT TGC GGG CGC	72
5wf-005 1FG	TAG GGC GCG AAG AAA GCG AAA GGA TTT TAT CGG CAA AAT CCC TTT GAT GGT GGT TCC GAA TTT TCC GCT TTC	72
5wf-006 1HI	CAG TCG GGG CGT TGC GCT CAC TGC TTT TCG TTG TAA AAC GAC GGG TTT TCC CAG TCA CGA TTT TAG GGG ACG	72
5wf-007 1JK	ACG ACA GTT GCA TCT GCC AGT TTG TTT TAG CCC CAA AAA CAG GAG GTT GAT AAT CAG AAA TTT TTC ATT GCC	72
5wf-008 1LM	TGA GAG TCT ACA AAG GCT ATC AGG TTT TGT CAA ATC ACC ATC AAA GAA AGG CCG GAG ACA TTT TCA AGG ATA	72
5wf-009 1NO	AAA ATT TTA GCC TTT ATT TCA ACG TTT TTC TAC TAA TAG TAG TAA AAA GGT GGC ATC AAT	60
5wf-010 2AB	GTC TGA AAC ACG ACC AGT AAT AAA TGC GCG AAC TGA TAG CAC CAC CAG CAG AAG ATA AAA CAG A	64
5wf-011 2CD	AGG GAT TTG TGT TTT TAT AAT CAG CGC AAA TTA ACC GTT GCT TGC CTG AGT AGA AGG CTC AAT C	64
5wf-012 2EF	AAG GAA GGT GGC AAG TGT AGC GGT TTA ATG CGC CGC TAC ATA TAA CGT GCT TTC CTC CGA TTA A	64
5wf-013 2GH	CAT TAA TTA AAC CTG TCG TGC CAG AGG CGA AAA TCC TGT TAT AAA TCA AAA GAA TAT GGC GAG A	64
5wf-014 2IJ	CGT AAC CGA TCG GCC TCA GGA AGA GTT GGG TAA CGC CAG GCC AGT GCC AAG CTT GCC TAA CTC A	64
5wf-015 2KL	GAG AGA TCT GGA GCA AAC AAG AGA CAA TCA TAT GTA CCC CAG ATT GTA TAA GCA AAG GGC GCA T	64
5wf-016 2MN	GCG GGA GAT AGA ACC CTC ATA TAT AAG ATT CAA AAG GGT GTA TGA TAT TCA ACC GTC TAT TTT T	64
5wf-017 2OP	CTA AAG TAG GAA GTT TCA TTC CAT TTT GGG GCG CGA GCT GGC ATT AAC ATC CAA TAA TAC TTT T	64
5wf-018 3AB	GGT GAG GCG GCT ATT AGT CTT TAA AGG GAC ATT CTG GCC AAT ACC TAC ATT TTG ACA ACT CAA A	64
5wf-019 3C	CTA TCG GCA AGA GTC TGT CCA TCA TGA GGC CA	32
5wf-020 3D	CCG AGT AAG GAG CTA AAC AGG AGG CGT TAG AA	32
5wf-021 3E	TCA GAG CGA CCA CAC CCG CCG CGC CAC GCT GC	32
5wf-022 3F	GCG TAA CCG GAA AGC CGG CGA ACG GCC CGA GA	32
5wf-023 3G	TAG GGT TGG CTG GTT TGC CCC AGC CTG CAT TA	32
5wf-024 3H	ATG AAT CGG TGC CTA ATG AGT GAG ATG CCT GC	32
5wf-025 3I	AGG TCG ACG CTG CAA GGC GAT TAA TCG CAC TC	32
5wf-026 3J	CAG CCA GCT CAC GTT GGT GTA GAT TAT TTA AA	32
5wf-027 3K	TTG TAA ACC GTA AAA CTA GCA TGT ATC GAT GA	32

5wf-028 3L	ACG GTA ATA TGC CGG AGA GGG TAG TCT AGC TG	32
5wf-029 3M	ATA AAT TAG AGT AAT GTG TAG GTA TTT AAA TG	32
5wf-030 3N	CAA TGC CTA CAT TAT GAC CCT GTA AAT CAT AC	32
5wf-031 3OP	AGG CAA GGT TTA GCT ATA TTT TCA ATA ACA GTT GAT TCC CGC TCA ACA TGT TTT AAA TAT GCA A	64
5wf-032 4A	TTT TGA ATG GTC AGT ATT AAC ACC GCC TGC AAC AGT GCC A	40
5wf-033 4B	CTT GCT GGT AAT ATC CAA AAA CGC TCA TGG AAA CAG AGA TAG AAC CCT GAC AAT AT	56
5wf-034 4G	CGG TCC ACA GTG TTG TTC CAG TTT ATT TAG AGC TTG ACG G	40
5wf-035 4H	AGC CTG GGG CCA ACG CGC GGG GAG GCA GCA AG	32
5wf-036 4I	GGG GAT GTT CTA GAG GAT CCC CGG AAG TGT AA	32
5wf-037 4J	GTT AAT ATT TTG TTA AAC CGT AAT GGG ATA GGT TTC CGG CAC CGC TTC GGC GAA AG	56
5wf-038 4O	ATA ACC TGC AAA GAA TTA GCA AAA ATC GGT TGT ACC AAA A	40
5wf-039 4P	CTT AGA GCT TAA TTG CTG AAT ATA ATG CTG TAA ATT CTG CGA ACG AGT AAT GGT CA	56
5wf-040 5-6A	AAG GAA TTG TCA GTT GGC AAA TCA AAG AAT ACG TGG CAC ATC TGA CCT GAA AGC GTA CAG TTG A	64
5wf-041 5-6B	ATT ACC GCT AAT TTT AAA AGT TTG TTT GCC CGA ACG TTA TCA GCC ATT GCA ACA GGA GAA CAA T	64
5wf-042 5F	TCG GAA CCC TAA AGG GAG CCC CCG GGA ACA AG	32
5wf-043 5G	AGT CCA CTT GGC CCT GAG AGA GTT AGG CGG TT	32
5wf-044 5H	TGC GTA TTA CGA GCC GGA AGC ATA GTA CCG AG	32
5wf-045 5I	CTC GAA TTC GCT ATT ACG CCA GCT TGG TGC CG	32
5wf-046 5J	GAA ACC AGA ACA AAC GGC GGA TTG AAT TCG CA	32
5wf-047 5-6N	TAA AGC CTA ATC CCC CTC AAA TGC TCA TAA ATA TTC ATT GCA GAG CAT AAA GCT AAT TAA GCA A	64
5wf-048 5-6O	TCA AAA AGT AGT CAG AAG CAA AGC TTA GAT ACA TTT CGC AAG ATT TAG TTT GAC CAG GAT TGC A	64
5wf-049 6G	TCA CCG CCA TTA AAG AAC GTG GAC GCA CTA AA	32
5wf-050 6H	CAC CGA CTC CAA AGA CAA AAG GGC GAT TAA GA	32
5wf-051 6I	GGC CTC TTC GTA ATC ATG GTC ATA CAA TTC CA	32
5wf-052 6J	TCC GTG GGG CAA AGC GCC ATT CGC TCG GTG CG	32
5wf-053 6K	TTA AAT TTT TGT TAA ATC AGC TCA TCG GAT TC	32
5wf-054 7A	CGC TGA GAG CCA GCA GCA AAT GAA AAA TCT AAC CTC AAT C	40
5wf-055 7B	AAT ATC TGG AGG AAG GTT ATC TAA AAC TCG TAT TAA ATC CAG TAA CAT TAT CAT TT	56
5wf-056 7G	GTC GAG GTG CCG TAA ATC CAA CGT CAA AGG GCA GAC GGG C	40
5wf-057 7H	AAC AGC TGT TTC TTT TCA CCA GTG AAT TGT TA	32
5wf-058 7I	TCC GCT CAG CTG TTT CCT GTG TGA CTG TTG GG	32
5wf-059 7J	AAG GGC GAC ATT CAG GCT GCG CAA AGC GAG TAA CAA CCC GTT TTT TAA CCA ATA GG	56
5wf-060 7O	CAA TAC TGC GGA ATC GTT TAA ACA GTT CAG AAT TTA CCC T	40
5wf-061 7P	GAC TAT TAA TTA AGA GGA AGC CCG TCC TTT TGA TAA GAG GTC ATT TTT GCG GAT GG	56
5wf-062 8-9A	AGA AAA CTA CCT CAA ATA TCA AAC AGC ATC ACC TTG CTG ATT TTC AAA TAT ATT TTA GAA CGC G	64
5wf-063 8B	AAA GAA ACT TAC AAA CAA TTC GAC AAT ATC TTT AGG AGC ACT AAC AAC	48
5wf-064 8C	CAT ATT CCC ACC AGA AGG AGC GGA TGC GGA AC	32
5wf-065 8D	CAA AAT TAT GGA AGG GTT AGA ACC ATT ATC AT	32
5wf-066 8E	AAA TTG CGT TTG CAC GTA AAA CAG TAC CAT AT	32

5wf-067 8F	GAA AAA CCG TCT ATC AAA TCA AGT TTT TTG GGA AAT AAA G	40
5wf-068 8K	CTA ACG GAA ACG CCA TCA AAA ATA ATC AAC ATT AAA TGT G	40
5wf-069 8L	TAC GAG GCA CAA CAT TAT TAC AGG AAA ACG AA	32
5wf-070 8M	CCC TCG TTA TAG TAA GAG CAA CAC AAA GGA AT	32
5wf-071 8N	ATA GCG TCT ACC AGA CGA CGA TAA TAT CAT AA	32
5wf-072 8O	CAA ATA TCA ATC AAA AAT CAG GTC AAC GAG AAT GAC CAT ATA GAC TGG	48
5wf-073 8-9P	ATT CGA GCA CAG GTC AGG ATT AGA GAG TAC CTT TAA TTG CAA AGA CTT	48
5wf-074 9BC	TAA TAG ATT TAG AAG TAT TAG ACT GAA ACA GTA CAT AAA TAT TAC CTT	48
5wf-075 9D	TTT TAA TGT GAT TAT CAG ATG ATG TTA TAC TTC TGA ATA AAG TTA CAA	48
5wf-076 9E	AAT CGC GCA TTG CTT TGA ATA CCA TAG ATT TTC AGG TTT AAC GTC AGA	48
5wf-077 9-10F	ATC ACC CAG GGC GAT GGC CCA CTA GAG AGA TAA CCC ACA ATT GAG CGC TAA TAT CAC GTG AAC C	64
5wf-078 9-10J	CTA TTT CGG TAT AAA CAG TTA ATG CCT GTA GCC AGC TTT CAT TCG CGT CTG GCC TTC CCC CTG C	64
5wf-079 9KL	ACG TTA ATT AGA AAG ATT CAT CAG CAG ATA CAT AAC GCC AAG AAC GAG	48
5wf-080 9MN	TAG TAA ATC CTG ACG AGA AAC ACC AAA CCA AAA TAG CGA GGT AAT AGT	48
5wf-081 9O	AAA ATG TTC AGA CGG TCA ATC ATA TAG CCG GAA CGA GGC GGC GTT TTA	48
5wf-082 10-11A	CAA GAC AAA GTT AAT TTC ATC TTC TGA CCT AAA TTT AAT GTA AAT GCT	48
5wf-083 10BC	GTG AGT GAT AAT ACA TTT GAG GAT TAG AGC CGT CAA TAG ATC CAA TCG	48
5wf-084 10D	TGT TTG GAG CAA TTC ATC AAT ATA TAA CAA TTT CAT TTG ACA ATA TAT	48
5wf-085 10E	TGA ATA TAA TAA CGG ATT CGC CTG AGA GGC GAA TTA TTC AAT CCT GAT	48
5wf-086 10KL	AAC TAA TGT TGA GAT TTA GGA ATA TCA GGA CGT TGG GAA GAA AAA TCT	48
5wf-087 10MN	GCA AAA GAA GTG AAT AAG GCT TGC TGG GCT TGA GAT GGT TCC ACA TTC	48
5wf-088 10O	ATG TTA CTA GGG AAC CGA ACT GAC AGT TTT GCC AGA GGG GAG GCT TTT	48
5wf-089 10-11P	GAA CCA GAA CAC TCA TCT TTG ACC AAA AGA ATA CAC TAA ACC GGA AGC AAA CTC CAT TCA AAG C	64
5wf-090 11B	GAT GCA AAA ATA GTG AAT TTA TCA GAC GCT GAG AAG AGT CAT AAC CTT	48
5wf-091 11C	GCT TCT GTC AAA ATT AAT TAC ATT ACA AAC AT	32
5wf-092 11D	CAA GAA AAA AAA GAA GAT GAT GAA TTT CAA TT	32
5wf-093 11E	ACC TGA GCT ACA TCG GGA GAA ACA CAG TAA CA	32
5wf-094 11F	GTA CCT TTC AAA GTC AGA GGG TAA GAA TTG AGT TAA GCC C	40
5wf-095 11K	TTG AGT AAC AGT GCC CGA ACC TAT TAT TCT GAT GGC TCA T	40
5wf-096 11L	TAT ACC AGA TGC GAT TTT AAG AAC CAT TGT GA	32
5wf-097 11M	ATT ACC TTT AAT TTC AAC TTT AAT ACA AAG CT	32
5wf-098 11N	GCT CAT TCT ACC CAA ATC AAC GTA AGA ACC GG	32
5wf-099 11O	ATA TTC ATC AAC TTT GAA AGA GGA GTG TCG AAA TCC GCG ACC TGC TCC	48
5wf-100 12A	ACT ATA TGG TTT GAA ATA CCG ACC GTG TGA TAA ATA AGG C	40
5wf-101 12B	AAA TCG TCG CTA TTA ACG ATA GCT TAG ATT AAA AAT CAT AGG TCT GAG GTT ATA TA	56

5wf-102 12G	AGA AAC GCA ATA ATA AGA GCA AGA AAC TGA ACA CCC TGA A	40
5wf-103 12H	AAG GCC GGA AAG ACA CCA CGG AAT CAT ATA AA	32
5wf-104 12I	CAG AGC CAA AAC GTC ACC AAT GAA CCA TTA GC	32
5wf-105 12J	AAC ATG AAA GTA TTA ATA ACG GGG TCA GTG CCC CAC CCT CAG AGC CGC GCC ACC CT	56
5wf-106 12O	GAT AAA TTC AGA TGA ACG GTG TAC AAG AGT AAT CTT GAC A	40
5wf-107 12P	CTA CGA AGG CAC CAA CCT AAA ACG AAA GAG GCC CCA GCG ATT ATA CCA CAT CGC CT	56
5wf-108 13-14A	GAG CCA GTT GTA ATT TAG GCA GAG CTC CGG CTT AGG TTG GAG ACT ACC TTT TTA ACG CAT TTT C	64
5wf-109 13-14B	CCC TTA GAT TTA CGA GCA TGT AGA TAA TAT CCC ATC CTA AAT CCT TGA AAA CAT AGT TAA TTT T	64
5wf-110 13F	GGA AGC GCA TTA GAC GGG AGA ATT AAC AAT GA	32
5wf-111 13G	AAT AGC AAT ACA TAA AGG TGG CAA AAG TTT AT	32
5wf-112 13H	TTT GTC ACA CCA GTA GCA CCA TTA ACC ATC GA	32
5wf-113 13I	TAG CAG CAC GCC ACC CTC AGA ACC CAC CAG AA	32
5wf-114 13J	CCA CCA CCT ACT GGT AAT AAG TTT GAG GCT GA	32
5wf-115 13-14N	AAA AGG AGG AAA ATC TCC AAA AAA CTG GCT GAC CTT CAT CAG ACC AGG CGC ATA GGA AGG CTC C	64
5wf-116 13-14O	ACA AAG TAG CCG CTT TTG CGG GAT TTG CAG GGA GTT AAA GCA ACG GAG ATT TGT ATA GCG CGA A	64
5wf-117 14G	AAA ATA CAT AGC TAT CTT ACC GAA AAA AAC AG	32
5wf-118 14H	GCA AAA TCA ATC AAT AGA AAA TTC AAA CGT AG	32
5wf-119 14I	CTC AGA GCC CGT AAT CAG TAG CGA TAG AGC CA	32
5wf-120 14J	CAG GAG TGA GAG CCG CCG CCA GCA CCG CCT CC	32
5wf-121 14K	GAC TCC TCA AGA GAA GGA TTA GGA TGA TGA TA	32
5wf-122 15A	GTT AAA TAA GAA TAA ACA CCG GAA TCA TAA TTT TTA ACA A	40
5wf-123 15B	CGC CAA CAA ATA AGA GAA TAT AAA TCC TGA ACA AGA AAA AAA CCA ATC AAT AAT CG	56
5wf-124 15G	ACA GAG AGA ATA ACA TGC CCT TTT TAA GAA AAT ACG CAG T	40
5wf-125 15H	ATG TTA GCA TAT GGT TTA CCA GCG TGA GCC AT	32
5wf-126 15I	TTG GGA ATC AGA ATC AAG TTT GCC AGA GCC AC	32
5wf-127 15J	CAC CGG AAT TGA CAG GAG GTT GAG CGT CAT ACA TGG CTT TTT AGC GGG GTT TTG CT	56
5wf-128 15O	ATA ATT TTT TCA CGT TCC TTT AAT TGT ATC GGA TTC GGT C	40
5wf-129 15P	GCT GAG GCC GTC ACC CTC AGC AGC TTT CCA TTA AAC GGG TAA AAT ACG TAA TGC CA	56
5wf-130 16AB	TAG ATA AGG TAC CGA CAA AAG GTA ATT GAG AAT CGC CAT AAC TAG AAA AAG CCT GTT TAG TAT C	64
5wf-131 16C	GGA ATC ATG CTG TCT TTC CTT ATC ATC AAC AA	32
5wf-132 16D	CGG GAG GTT ACC GCG CCC AAT AGC TCA TCG TA	32
5wf-133 16E	AGA GCC TAT TTG AAG CCT TAA ATC CCG ACT TG	32
5wf-134 16F	CAG CCT TTA TTT GCC AGT TAC AAA GTC TTT CC	32
5wf-135 16G	CTC CTT ATG TAA GCA GAT AGC CGA GAA AAT AG	32
5wf-136 16H	CAC AAC ATG GGC GCC AGG GTG GTT ATT GCC CT	32
5wf-137 16I	CCG GAA CCT TTA GCG TCA GAC TGT ATC ACC GT	32
5wf-138 16J	CCA GTA AGG CAG GTC AGA CGA TTG CAA AAT CA	32
5wf-139 16K	GCC ACC CTC AGT ACC AGG CGG ATA TTA CCG TT	32
5wf-140 16L	ACA CTG AGC AGA ACC GCC ACC CTC TTA GTA CC	32
5wf-141 16M	CCA GAC GTT TTC GTC ACC AGT ACA GTA CCG TA	32

5wf-142 16N	TGC GAA TAT AGT AAA TGA ATT TTC TCG TCT TT	32
5wf-143 16OP	TGA GGA AGG AAA GAC AGC ATC GGA CAC GCA TAA CCG ATA TTT TAT CAG CTT GCT TTA AAG GAA T	64
5wf-144 17AB	ATA TGC GTT CAA CAG TAG GGC TTA AAG TAA TTC TGT CCA GCA GAA CGC GCC TGT TTA TTC CAA G	64
5wf-145 17CD	AAC GGG TAC AAG CCG TTT TTA TTT AAG CAA ATC AGA TAT ACG TTT TAG CGA ACC TCA AGA TTA G	64
5wf-146 17EF	TTG CTA TTA CCA ACG CTA ACG AGC ATA AAC AGC CAT ATT AGT TTA ACG TCA AAA ATA CAA AGT T	64
5wf-147 17GH	ACC AGA AGC CAA AAG AAC TGG CAT GAC ATT CAA CCG ATT GTC ATT AAA GGT GAA TTA GCG CGT T	64
5wf-148 17IJ	TTC ATC GGG CCA TCT TTT CAT AAT GCC TTG ATA TTC ACA AAG CGC AGT CTC TGA ATA GTG CCG T	64
5wf-149 17KL	CGA GAG GGA CCG TAC TCA GGA GGT AGA ACC GCC ACC CTC ACC CAA TAG GAA CCC ATA ACT ACA A	64
5wf-150 17MN	CGC CTG TAA CGA TCT AAA GTT TTG TGT ATG GGA TTT TGC TAT AGA AAG GAA CAA CTC GAG GTG A	64
5wf-151 17OP	ATT TCT TAG ACA ACA ACC ATC GCC ACG AGG GTA GCA ACG GCT TTG AGG ACT AAA GAC TTT TTC A	64
5wf-152 18A	AAT AAA CAT TTT AGT ATA AAG CCA ACG CTA TAC AAA TTC TTA CC	44
5wf-153 18BC	ATC CGG TAT TTT ACT CAT CGA GAA CAA GTT AAA CCA AGT ACC GCT TTT ACA TGT TCA GCT AAT GAC GAC GAC	72
5wf-154 18DE	AAT CCA AAT TTT TTT TAT CCT GAA TCT TTT GCA CCC AGC TAC AAT TTT TTC TAA GAA CGC GAG GGA AGG CTT	72
5wf-155 18FG	AAG GTA AAT TTT AAT AAT AAC GGA ATA CGA AAC CGA GGA AAC GCT TTT TAA GAA ACG ATT TTT TTT TAT CCC	72
5wf-156 18HI	ATC CTC ATT TTT CCC CTT ATT AGC GTT TCA TTT TCG GTC ATA GCT TTT TAT TGA CGG AAA TTA TAG GGA GGG	72
5wf-157 18JK	ACC CTC ATT TTT CCG GAA TAG GTG TAT CTT GAT ATA AGT ATA GCT TTT TAA AGC CAG AAT GGA AAC AAA TAA	72
5wf-158 18LM	TTC AAC AGT TTT CCT CAT AGT TAG CGT AGC ATT CCA CAG ACA GCT TTT TTT CAG GGA TAG CAA GGA GCC ACC	72
5wf-159 18NO	TAG TTG CGC CGA CAA TAA CAG CTT GAT ACC GAT TTT TTT CAG CGG AGT GAG AAA ACA ACT	60

Table S-1. Sequences of the 159 complementary oligos.

The DNA nanostructures were made by mixing together 2 μL M13mp18 DNA (10 nM), 5μL oligonucleotide mix (each oligo 200 nM), 2 μL origami buffer 10x (20 mM $MgCl_2$, 100 mM Tris-HCl, pH=7.6), and 11 μL deionised MilliQ water. EDTA was omitted from the origami buffer for the whole of this study (normally included at 1mM), so as not to interfere with the bacterial populations. The mixture was annealed from 85 to 25 °C at a rate of −1.0 °C/min. After annealing, excess oligonucleotides were removed using a Micro Biospin column (Bio-Rad) packed with Sephacryl S-300 (GE Healthcare).

1.2 Modifications to the 5-well frames

The following oligonucleotides were modified to carry aptamers that can bind *E.coli* and *B.subtilis*[2]*.* The aptamers sequence is CAT ATC CGC GTC GCT GCG CTC AGA CCC ACC ACC ACG CAC C (in red in the table below).

Sequence Name	Sequence	Bases
5wf-009 1NO-apt	AAA ATT TTA GCC TTT ATT TCA ACG TTT TTC TAC TAA TAG TAG TAA AAA GGT GGC ATC AAT TTT TT C ATA TCC GCG TCG CTG CGC TCA GAC CCA CCA CCA CGC ACC	105
5wf-015 2KL-apt	GAG AGA TCT GGA GCA AAC AAG AGA CAA TCA TAT GTA CCC CAG ATT GTA TAA GCA AAG GGC GCA TTT TTT CAT ATC CGC GTC GCT GCG CTC AGA CCC ACC ACC ACG CAC C	109
5wf-159 18NO-apt	TAG TTG CGC CGA CAA TAA CAG CTT GAT ACC GAT TTT TTT CAG CGG AGT GAG AAA ACA ACT TTT TT C ATA TCC GCG TCG CTG CGC TCA GAC CCA CCA CCA CGC ACC	105
5wf-001 1A-apt	TAA AAA TAC CGA ACG ACC TAA AAC ATC GCC ATG CAG ATT CTT TTT CAT ATC CGC GTC GCT GCG CTC AGA CCC ACC ACC ACG CAC C	85
5wf-023 3G-apt	TAG GGT TGG CTG GTT TGC CCC AGC CTG CAT TAT TTT T CA TAT CCG CGT CGC TGC GCT CAG ACC CAC CAC CAC GCA CC	77
5wf-043 5G - apt	AGT CCA CTT GGC CCT GAG AGA GTT AGG CGG TTT TTT T CA TAT CCG CGT CGC TGC GCT CAG ACC CAC CAC CAC GCA CC	77
5wf-045 5I - apt	CTC GAA TTC GCT ATT ACG CCA GCT TGG TGC CGT TTT T CA TAT CCG CGT CGC TGC GCT CAG ACC CAC CAC CAC GCA CC	77
5wf-075 9D-apt	TTT TAA TGT GAT TAT CAG ATG ATG TTA TAC TTC TGA ATA AAG TTA CAA TTT TT C ATA TCC GCG TCG CTG CGC TCA GAC CCA CCA CCA CGC ACC	93
5wf-111 13G-apt	AAT AGC AAT ACA TAA AGG TGG CAA AAG TTT ATT TTT T CA TAT CCG CGT CGC TGC GCT CAG ACC CAC CAC CAC GCA CC	77
5wf-113 13I - apt	TAG CAG CAC GCC ACC CTC AGA ACC CAC CAG AAT TTT T CA TAT CCG CGT CGC TGC GCT CAG ACC CAC CAC CAC GCA CC	77
5wf-136 16H-apt	CAC AAC ATG GGC GCC AGG GTG GTT ATT GCC CTT TTT T CA TAT CCG CGT CGC TGC GCT CAG ACC CAC CAC CAC GCA CC	77
5wf-138 16J - apt	CCA GTA AGG CAG GTC AGA CGA TTG CAA AAT CAT TTT T CA TAT CCG CGT CGC TGC GCT CAG ACC CAC CAC CAC GCA CC	77
5wf-152 18A-apt	AAT AAA CAT TTT AGT ATA AAG CCA ACG CTA TAC AAA TTC TTA CCT TTT T CA TAT CCG CGT CGC TGC GCT CAG ACC CAC CAC CAC GCA CC	89
5wf-079 9KL-apt	ACG TTA ATT AGA AAG ATT CAT CAG CAG ATA CAT AAC GCC AAG AAC GAG TTT TT C ATA TCC GCG TCG CTG CGC TCA GAC CCA CCA CCA CGC ACC	93

Table S-2. Sequences of the 14 aptamer-modified oligos.

The following oligonucleotides were functionalised with Alexa 647 molecules.

5wf-025 3I 647	/5Alex647N/TTT TTA GGT CGA CGC TGC AAG GCG ATT AAT CGC ACT C	37
5wf-126 15I 647	/5Alex647N/TTT TTT TGG GAA TCA GAA TCA AGT TTG CCA GAG CCA C	37
5wf-083 10BC 647	/5Alex647N/TTT TTG TGA GTG ATA ATA CAT TTG AGG ATT AGA GCC GTC AAT AGA TCC AAT CG	53
5wf-081 9O 647	/5Alex647N/TTT TTA AAA TGT TCA GAC GGT CAA TCA TAT AGC CGG AAC GAG GCG GCG TTT TA	53

Table S-3. Sequences of the 4 Alexa 647-modified oligos.

The following oligonucleotides were functionalised to carry biotin:

5wf-057 7H-bio	AAC AGC TGT TTC TTT TCA CCA GTG TTT TT/3Bio/	29
5wf-066 8E-bio	AAA TTG CGT TTG CAC GTA AAA CAG TTT TT/3Bio/	29
5wf-096 11L-bio	TAT ACC AGA TGC GAT TTT AAG AAC TTT TT/3Bio/	29
5wf-020 3D-bio	/5Biosg/TTT TTG GAG CTA AAC AGG AGG CGT TAG AA	29
5wf-028 3L-bio	/5Biosg/TTT TTA TGC CGG AGA GGG TAG TCT AGC TG	29

5wf-070 8M-bio	/5Biosg/TTT TTA TAG TAA GAG CAA CAC AAA GGA AT	29
5wf-092 11D-bio	/5Biosg/TTT TTA AAA GAA GAT GAT GAA TTT CAA TT	29
5wf-104 12I-bio	/5Biosg/TTT TTA AAC GTC ACC AAT GAA CCA TTA GC	29
5wf-133 16E-bio	/5Biosg/TTT TTT TTG AAG CCT TAA ATC CCG ACT TG	29
5wf-141 16M-bio	/5Biosg/TTT TTT TTC GTC ACC AGT ACA GTA CCG TA	29

Table S-4. Sequences of the 10 biotin-modified oligos.

Section 2. Imaging of the nanostructures

2.1 Atomic Force Microscopy (AFM)

Origami tiles were diluted ten times in origami buffer and 25 µl of the sample were deposited on freshly cleaved mica and incubated at room temperature for 10 minutes. The samples were then washed 5 times with 1 ml of origami buffer. The nanostructures were imaged in origami buffer, using a Dimension FastScan AFM microscope (Bruker). The probes used were FastScan-D probes (Bruker), with a resonant frequency of 90 kHz, a spring constant of 0.21 Nm^{-1} and a nominal tip radius of 8 nm.

DNA origami tiles were incubated with 0.17 µM Streptavidin (Sigma Aldrich) for 5 minutes at room temperature, after which they were filtered using a Millipore filter (Millipore, MA, USA) unit with molecular weight cut-off (MWCO) of 100 KDa to remove free protein. The tiles were then prepared for AFM imaging as described above.

To load the streptavidin functionalised origami tiles with the antimicrobial enzyme, the tiles were incubated with 1mg/ml biotinylated lysozyme (Chicken Lysozyme protein, Egg whites, GeneTex) for 10 minutes at room temperature after which they were filtered and imaged as above.

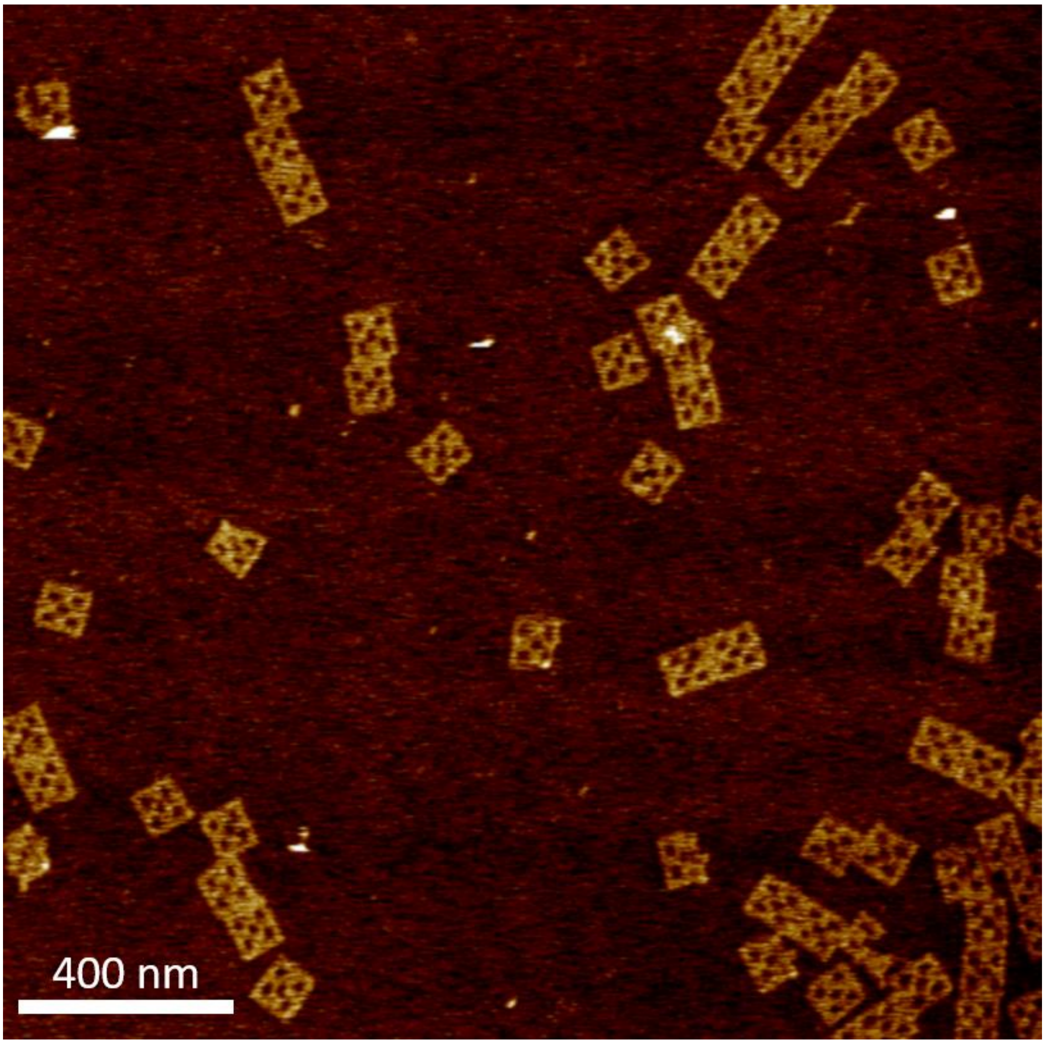

Figure S.1. Large field of view (2x2 μm) of DNA origami nanostructures.

2.2 AFM data analysis

Images of all datasets were plane-fitted using the speed-optimised plane correction function of the SPIP software (Image Metrology A/S, Hørsholm, Denmark), which fits each line in the horizontal axis to a polynomial equation. SPIP was also used for calculation of the volumes of proteins attached to the DNA origami tiles. The "inspection window" feature of SPIP was used to zoom into individual tiles and then the "circular area of interest" tool was used to allow the software to calculate only the volume of the protein rather than that of the whole tile, according to the following equation:

$$Z_{net\ volume} = Z_{material\ volume} - Z_{void\ volume}$$

where $Z_{material\ volume}$ is the volume of all pixels inside the shape's contour with a Z value greater than zero:

$$Z_{material\ volume} = \sum_{\{Z(x,y)\in shape|Z\geq 0\}} Z(x,y)dxdy$$

where dx and dy are the point spacings in the X and Y directions of the image, respectively. $Z_{void\ volume}$ is the volume of all pixels inside the shape's contour with a Z value lower than or equal to zero:

$$Z_{void\ volume} = \sum_{\{Z(x,y)\in shape|Z\leq 0\}} Z(x,y)dxdy$$

where dx and dy are the point spacing in the X and Y directions of the image, respectively.

For cross-sections of sample features, tile dimensions measurements, as well as for the 3D rendering of the images, Nanoscope 1.9 software (Bruker) was used. Volume histograms were drawn with bin widths chosen according to Scott's equation[3], using GraphPad Prism.

"Theoretical" molecular volumes of proteins based on molecular mass were calculated using the equation by Schneider et al[4]:

$$V=(M_0/N_0)(V_1 + dV_2)$$

where M_0 is the molecular mass, N_0 is Avogadro's number, V_1 and V_2 are the partial specific volumes of protein and water, respectively, and d is the extent of protein hydration. The partial specific volume of a typical protein (V_1) is considered to be 0.74 cm^3g^{-1}, and the extent of protein hydration (d) has been estimated to be 0.4 g of water/g of protein. The partial specific volume of water (V_2) is 1 cm^3g^{-1}

2.3 direct Stochastic Optical Reconstruction Microscopy (dSTORM)

Fluorescence microscopy experiments

The fluorescence microscopy experiments performed on the origami structures were conducted on a custom-built microscope based on an Olympus (Center Valley, PA) IX-73 frame with a 100x 1.49 NA oil objective lens (Olympus UAPON100XOTIRF) and a 647-nm laser (MPB Communications Inc. VFL-P-300-647-OEM1-B1). The samples were imaged in total internal reflection fluorescence (TIRF) mode and *d*STORM images collected on an Andor iXon Ultra 897 camera as described previously[5].

dSTORM on origami structures

16000 frames were acquired for the *d*STORM reconstructions, each recorded at 20 ms exposure time using an EM gain of 200 over a 256x256 pixel region. The pixel size in the image plane was measured to be 118 nm. The raw single molecule data sets were reconstructed using ThunderSTORM[6], and visualized as averaged shifted histograms with a magnification factor of 10. The peak-to-peak distance between the fluorophores tethered to the origami structures was measured by taking a cross-sectional profile in Fiji/ImageJ[7] between two bright spots in different regions of interest in the reconstructed image, and using a custom MATLAB (Natick, MA) script to measure the average distance between two peaks. Representative large fields of view can be seen in Figure S.2

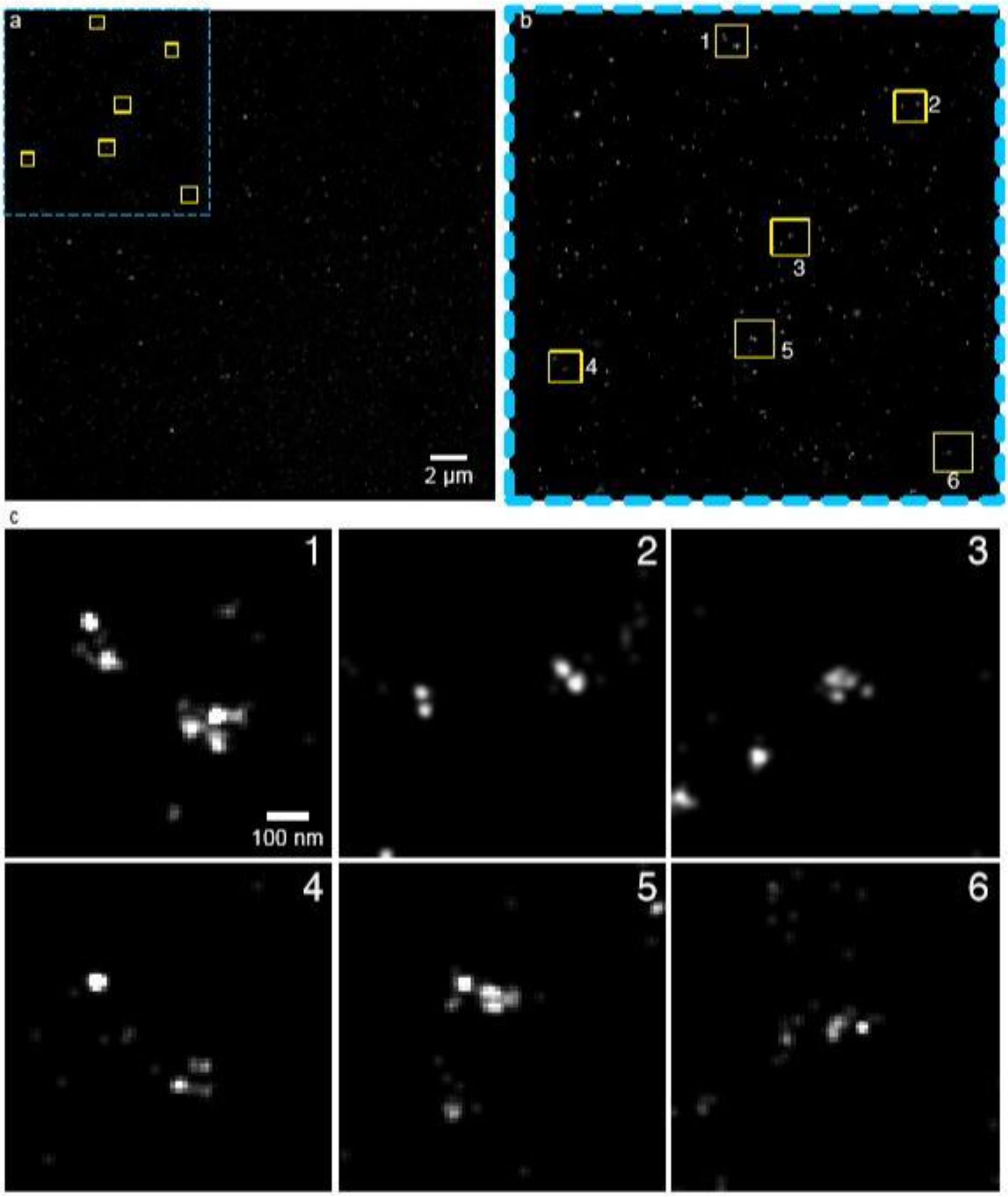

Figure S.2. *d*STORM reconstruction of origami structures labelled with AlexaFluor647 molecules attached to four anchor points. (a) Large FOV image of the *d*STORM reconstruction, with insets to highlight regions of interest containing origami structures. (b) shows a sub-panel from the large FOV image in (a) with 6 regions selected arbitrarily to highlight origami structures. (c) Zoomed-in insets of the origami structures highlighted in (b), with two (2), three (1,6), and four (3,4,5) detected fluorophore locations.

Section 3. Imaging of bacterial populations

3.1 Sample preparation for SIM

Each one of the bacterial strains was grown overnight (OD_{600} of ~1 in the case of *E. coli* and ~0.6 in the case of *B. subtilis*). Prior to SIM imaging the cultures were spun down and washed three times in origami buffer. The bacteria were then resuspended in 100 µl origami buffer. In the case of *B. subtilis*, the bacterial pellet was resuspended in 100 µl origami buffer containing 1 µg/ml Nile red dye (Sigma-Aldrich, 72485) to enable visualisation of the bacteria. This step was not required for *E. coli,* as the BL21(DE3) *E.coli* cells used in this experiment had been transformed with pUC19GFP plasmid, and express GFP, meaning no additional staining is required for this strain.

Subsequently, 10 µl of bacterial suspension were mixed with 10µl of DNA origami (final concentration ~10 nM) and incubated with shaking at room temperature for 15 mins. The bacteria were gently centrifuged and resuspended in origami buffer to remove excess or unbound origami tiles. 2µl of the sample were deposited on a glass coverslip and an agarose pad was positioned over the sample to prevent the bacteria from moving during imaging. Another coverslip was positioned on top to minimise drying of the agarose pads.

3.2 Structured Illumination Microscopy (SIM)

Images of the sample were collected using 3-color SIM for optical sectioning[8]. A ×60/1.2 NA water immersion lens (UPLSAPO 60XW, Olympus) focused the structured illumination pattern onto the sample, and the same lens was also used to capture the fluorescence emission light before imaging onto an sCMOS camera (C11440, Hamamatsu). The wavelengths used for excitation were 488 nm (iBEAM-SMART-488, Toptica), 561 nm (OBIS 561, Coherent), and 640 nm (MLD 640, Cobolt). Images were acquired using custom SIM software described previously[9].

An automated analysis routine for processing SIM images was written in MatLab. In order to quantify the overlap of the DNA origami (in magenta) with the bacterium body (in green), the code defines the percentage of the bacterium surface covered by DNA origami as the ratio between the number of overlapping pixels (pixels where both colours have non-zero intensity values) and the number of all pixels corresponding to single bacterium.

SIM imaging experiments were repeated three times, each time five fields of view were analysed to determine the DNA origami coverage of each of the two bacterial strains, with ~ 825 single bacteria for *E. coli* and ~750 single bacteria for *B. subtilis* analysed in total.

Representative large fields of view from where statistical analyses were obtained can be seen in Figures S.3 and S.4. The images in Figure 3 of the manuscript are subsets of those.

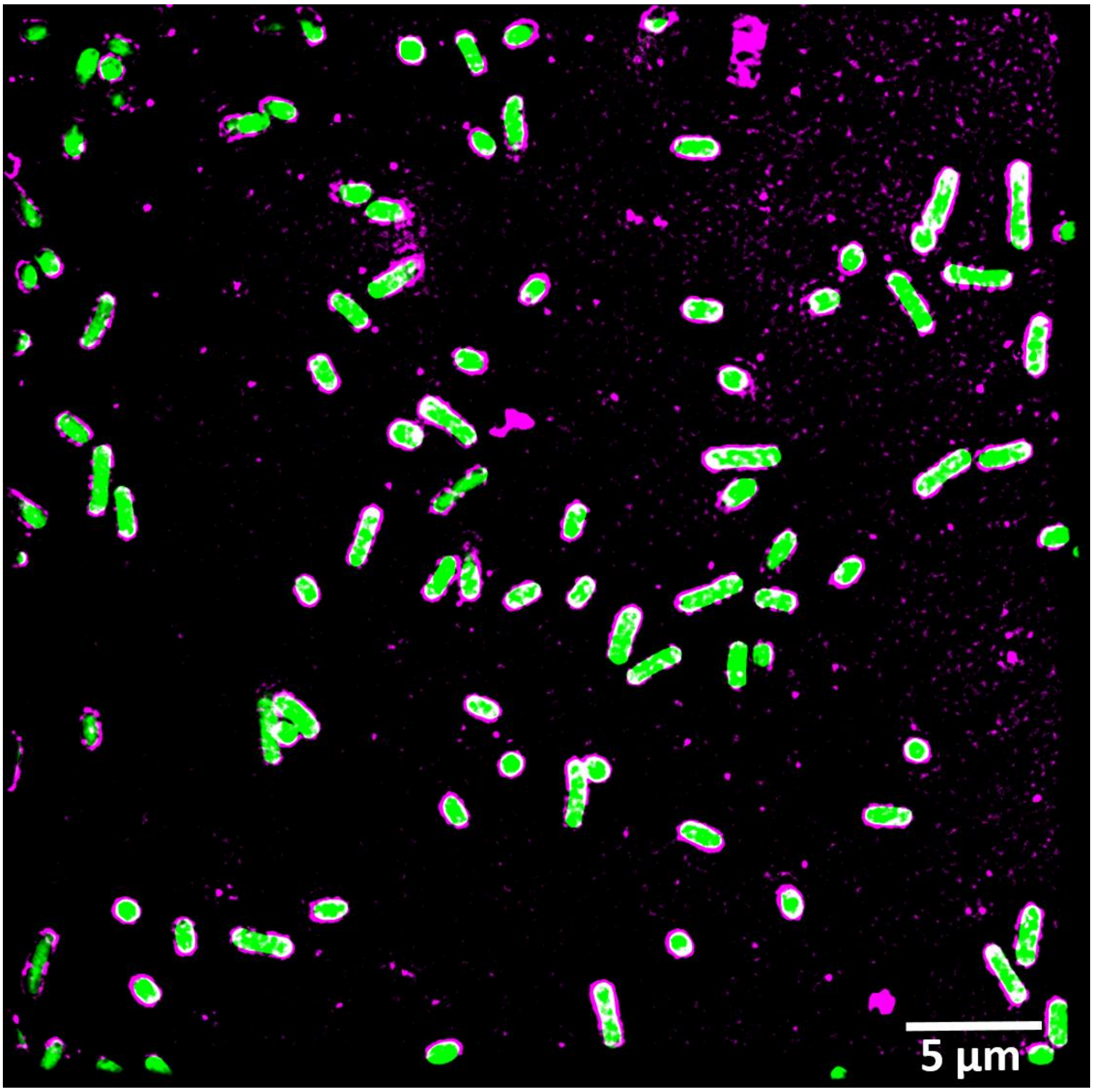

Figure S.3. Large field of view (42x42 µm) of E. coli (in green) decorated with DNA origami (in magenta). Overlap of the two colours is shown in white.

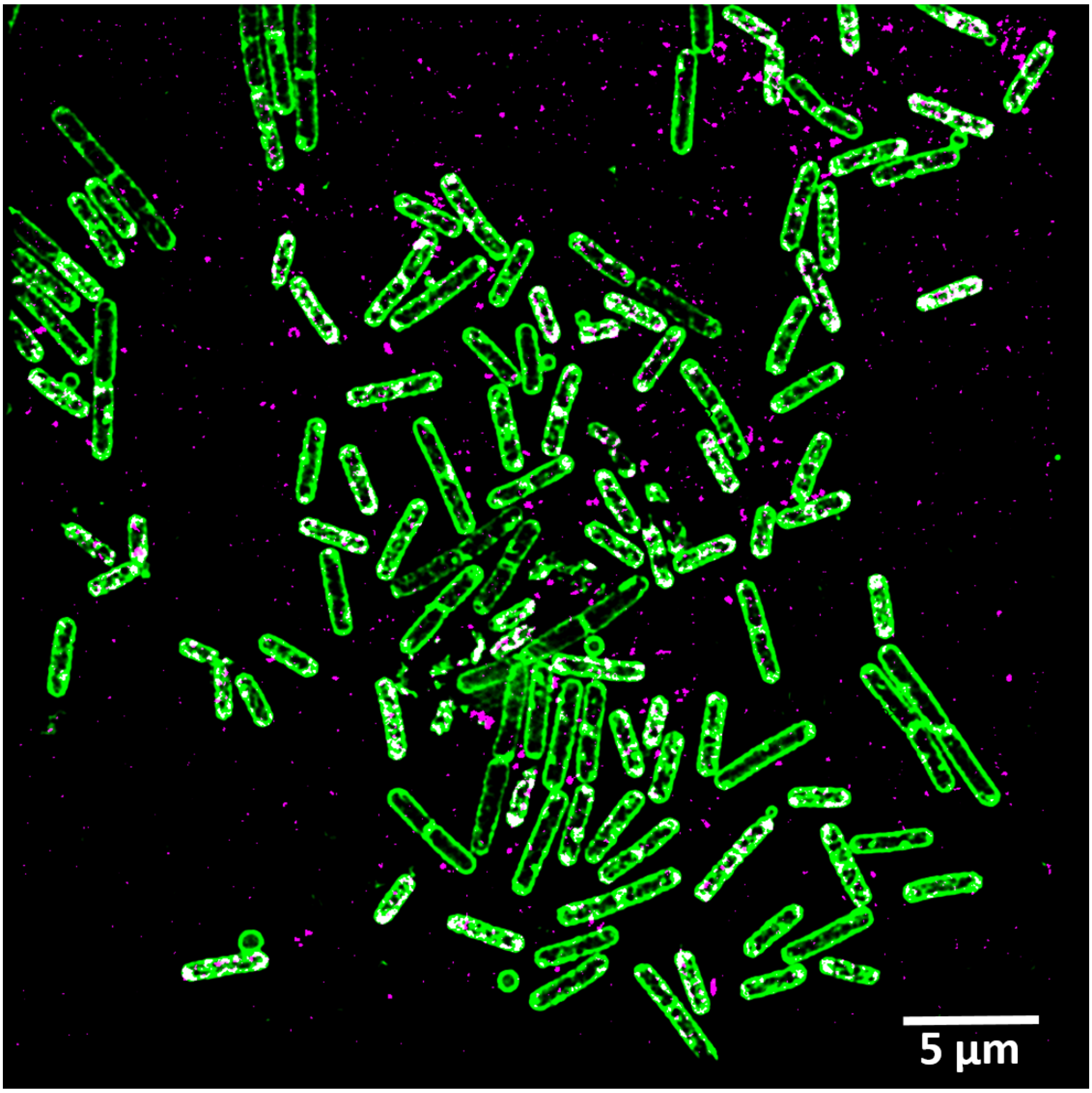

Figure S.4 Large field of view (42x42 µm) of B. subtilis (in green) decorated with DNA origami (in magenta). Overlap of the two colours is shown in white.

Section 4. Bacterial growth curves

Bacterial cell culture studies were conducted using *E. coli* BL21(DE3), expressing GFP and *B. subtilis* (BS168). All experiments were conducted in LB medium, supplemented with carbenicillin (100 µg/ml) for *E. coli* and chloramphenicol (25 µg/ml) for *B. subtilis*. Bacterial starter cultures were grown overnight, and the bacteria were then diluted 1:100 into 150µl LB, and grown over 16 hours in a shaking plate reader, at 37°C, with measurements taken every 5 minutes, in the following conditions:

E. coli	
**Sample 1**	**LB**
**Sample 2**	**LB + 10 nM DNA origami**
**Sample 3**	**LB + 0.3 µM free lysozyme**
**Sample 4**	**LB + 10 nM DNA origami carrying ~0.3µM lysozyme**
**Control 1**	**LB + 10 nM DNA origami w/o aptamers**
**Control 2**	**LB + Origami Buffer (10mM Tris, 2mM $MgCl_2$)**

Table S-5. Experimental conditions for *E. coli*

B. subtilis	
**Sample 1**	**LB**
**Sample 2**	**LB + 10 nM DNA origami**
**Sample 3**	**LB + 0.3 µM free lysozyme**
**Sample 4**	**LB + 10 nM DNA origami carrying ~0.3µM lysozyme**
**Control 1**	**LB + 10 nM DNA origami w/o aptamers**
**Control 2**	**LB + Origami Buffer (10mM Tris, 2mM $MgCl_2$)**

Table S-6. Experimental conditions for *B. subtilis*

The OD values at 600nm were collected and used for the creation of growth curves. For each condition, 9 individual growth curves were analysed and averaged. Individual growth curves were fitted in MATLAB using the curve fitting toolbox, to a re-parameterised Gompertz growth model[10], to extract growth rates.

DNA origami carrying lysozyme were prepared as described in Section 2.1 and added to the samples where appropriate.

The growth rates for *E. coli* and *B. subtilis* grown in the presence of DNA origami without aptamers are presented in Figure S.5:

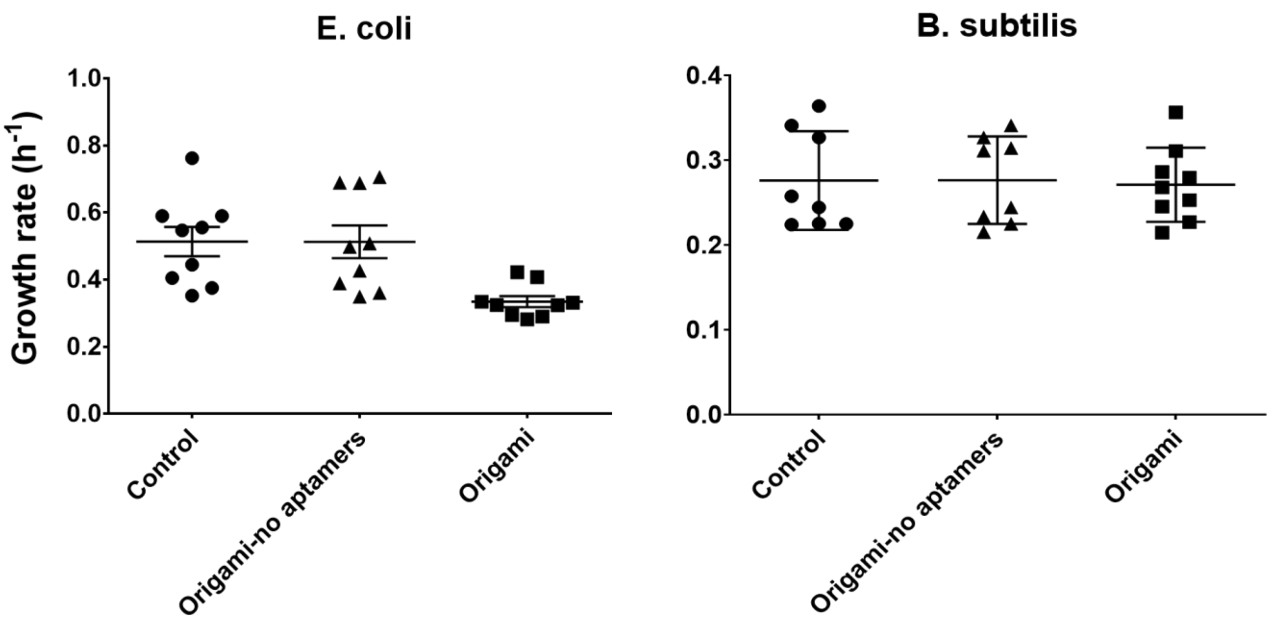

Figure S.5: Growth rates of *E. coli* and *B. subtilis* in the presence of DNA origami with and without aptamers.

To further assess the bacterial growth beyond the first eight hours, we plotted the OD values for the bacterial cultures between eight and sixteen hours of growth. No changes were observed, apart from the expected population decline due to the depletion of nutrients in the growth medium and the accumulation of toxic metabolic by-products.

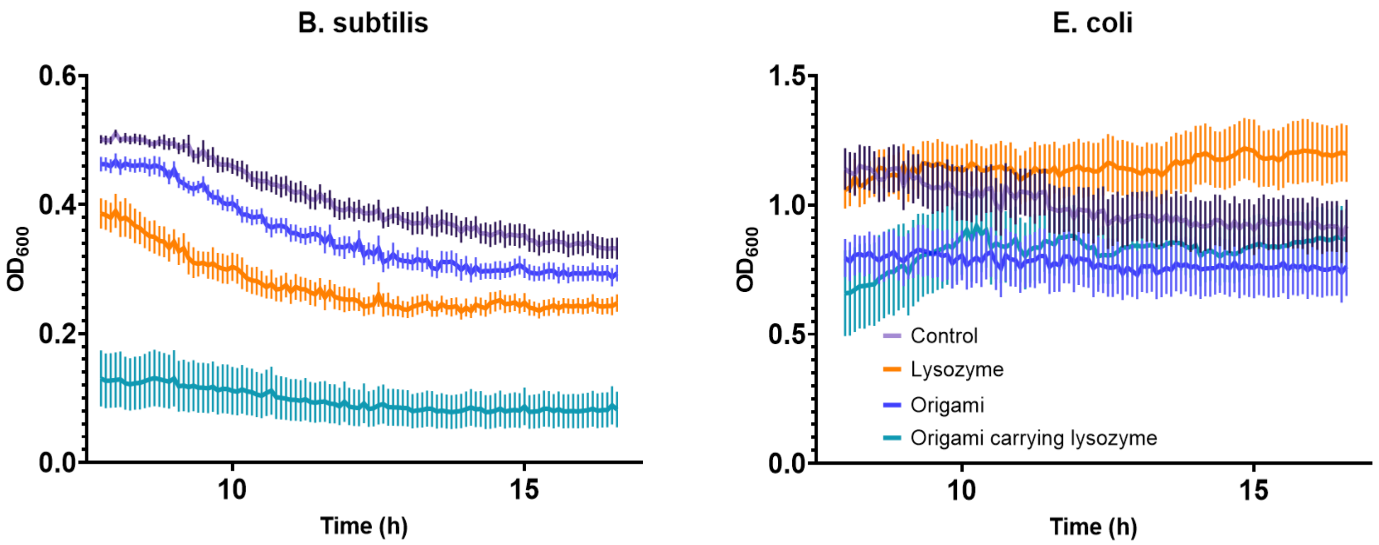

Figure S.6: Averaged growth curves for *B. subtilis* (n=9, left) and *E. coli* (n=9, right) show that the growth plateaus after eight hours of culture and no significant changes are observed beyond that point.

Section 5. Binding affinity of DNA nanostructures

We estimated the apparent dissociation constant K_d of the aptamer-functionalised nanostructures, to better understand their affinity for the bacterial targets and the impact of having many of them locally concentrated into a multivalent complex.

The aptamers used have a dissociation constant K_d of 27.2 nM for *E.coli* and 9.97 nM for *B.subtilis* according to Song *et al*[2]. Recently, Csizmar *et al.*[11] have used multivalent scaffolds to target tumour cells and have proposed the following equation to quantify the effect of the multiple valency, *N*, in a molecular scaffold, on its apparent affinity:

$$K = \frac{K_{d,1}}{N^2}$$

where $K_{d,1}$ is the affinity of a single-target ligand, and $K_{d,N}$ is the apparent affinity of the multiple-target ligand.

Our DNA nanostructure carries 14 aptamers; we thus obtain an apparent $K_{d,N}$ for *E. coli* and *B. subtilis* of 141 pM and 50 pM respectively.

Section 6. Cell viability assay

In order to explore the future potential of the DNA origami nanostructures to be used *in vivo* for selective bacterial targeting, we performed a mammalian cell viability assay. We used the CellTiter 96® AQueous One Solution Cell Proliferation Assay (Promega), to assess the effects of the DNA origami on mammalian cells. COS-7 cells were plated in a 96-well plate at concentration of 10,000 cells/well in 100µl of media (DMEM+10%FBS). 20µl of CellTiter 96® AQueous One Solution Reagent were added per well and the cells were incubated at 37°C for 2 hours in a humidified, 5% CO_2 atmosphere. After 2 h, the absorbance at 490nm was measured, using a 96-well plate reader. The measurements were performed in triplicates.

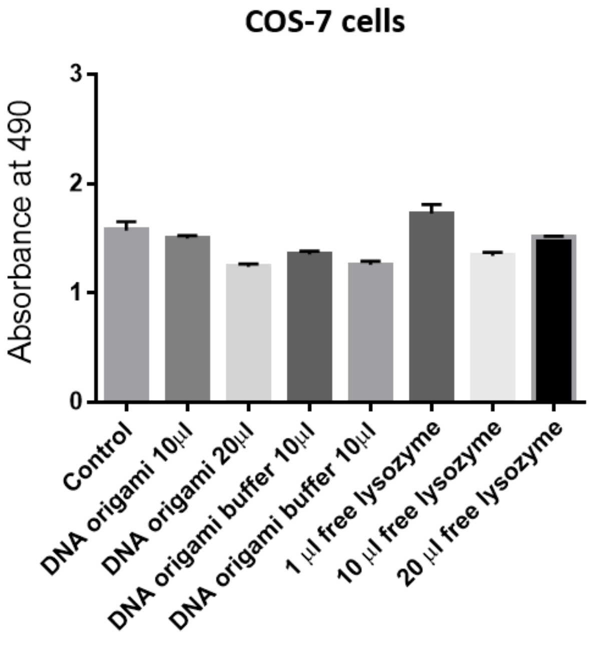

Figure S.7: Mammalian COS-7 cells are not affected by DNA origami

Section 7. Enzyme activity in the context of targeted delivery through DNA nanostructures

One question that is of great interest in the context of enzyme delivery through DNA nanostructures is the way in which the enzymatic activity is affected by binding of the enzyme to the DNA scaffold and how the high local enzyme concentrations afforded by the delivery vehicle affect the activity of the enzyme against its target.

In the case of lysozyme, it is possible that the loading of multiple enzymes on the same delivery platform leads to synergistic action that enhances its antimicrobial activity. Previous work has indeed indicated that the targeted delivery of several lysozyme molecules does increase antimicrobial activity locally. For example, it has been reported that all of Dextran-conjugated lysozyme[12] and chitosan-lysozyme[13] and selenium-lysozyme[14] nanoparticles loaded with increasing amounts of lysozyme increase activity of the enzyme. The activity of lysozyme has also been shown to increase through delivery via "Engineered Water Nanostructures"[15]. So overall there is plausible evidence that multiple loading sites provide cumulative benefits for antimicrobial applications, which will be explored in future work.

It is also possible that charge effects mediated by the DNA origami platform affect enzyme function. Although there is no available literature on lysozyme / DNA origami effects of this nature, an increased enzymatic activity was observed when other enzymes (i.e. not lysozyme) were coupled to DNA origami. For example, T. Morii's group used DNA origami to assemble ribulose biphosphate carboxylase/oxygenase (RuBisCO). They show that the enzymatic activity is retained upon binding and possibly enhanced[16]. Similarly, Zhao et al. showed that GOx/HRP enzyme pairs exhibit enhanced catalytic activity when bound to DNA nanocages[17], while Ora et al. report intact

activity of enzymes bound on DNA origami for delivery to mammalian cells[18]. Potentially these effects are indeed mediated by the charge of the DNA scaffold.

References

[1] T. Yoshidome, M. Endo, G. Kashiwazaki, K. Hidaka, T. Bando, H. Sugiyama, *J. Am. Chem. Soc.* **2012**, *134*, 4654–4660.

[2] M. Y. Song, D. Nguyen, S. W. Hong, B. C. Kim, *Sci. Rep.* **2017**, *7*, 43641.

[3] D. W. SCOTT, *Biometrika* **1979**, *66*, 605–610.

[4] S. W. Schneider, J. Lärmer, R. M. Henderson, H. Oberleithner, *Pflugers Arch. Eur. J. Physiol.* **1998**, DOI 10.1007/s004240050524.

[5] C. J. Rowlands, F. Ströhl, P. P. V. Ramirez, K. M. Scherer, C. F. Kaminski, *Sci. Rep.* **2018**, *8*, DOI 10.1038/s41598-018-24052-4.

[6] M. Ovesný, P. Křížek, J. Borkovec, Z. Švindrych, G. M. Hagen, *Bioinformatics* **2014**, *30*, 2389–2390.

[7] C. T. Rueden, J. Schindelin, M. C. Hiner, B. E. DeZonia, A. E. Walter, E. T. Arena, K. W. Eliceiri, *BMC Bioinformatics* **2017**, *18*, 529.

[8] F. Ströhl, C. F. Kaminski, *Methods Appl. Fluoresc.* **2015**, *3*, DOI 10.1088/2050-6120/3/1/014002.

[9] L. J. Young, F. Ströhl, C. F. Kaminski, *J. Vis. Exp.* **2016**, DOI 10.3791/53988.

[10] Z. M.H., J. I., R. F.M., van 'T R. K., *Appl. Environ. Microbiol.* **1990**, *56*, 1875–1881.

[11] C. M. Csizmar, J. R. Petersburg, T. J. Perry, L. Rozumalski, B. J. Hackel, C. R. Wagner, *J. Am. Chem. Soc.* **2019**, *141*, 251–261.

[12] J. Lee, I. Kim, S. Yeo, D. Kim, M. Kim, *Prev. Nutr. Food Sci.* **2018**, *23*, 60–69.

[13] C. N. Hernández-Téllez, F. J. Rodríguez-Córdova, E. C. Rosas-Burgos, M. O. Cortez-Rocha, A. Burgos-Hernández, J. Lizardi-Mendoza, W. Torres-Arreola, A. Martínez-Higuera, M. Plascencia-Jatomea, *3 Biotech* **2017**, *7*, 1–13.

[14] P. A. Tran, T. J. Webster, *Nanotechnology* **2013**, *24*, 155101.

[15] N. Vaze, G. Pyrgiotakis, L. Mena, R. Baumann, A. Demokritou, M. Ericsson, Y. Zhang, D. Bello, M. Eleftheriadou, P. Demokritou, *Food Control* **2019**, *96*, 365–374.

[16] H. Dinh, E. Nakata, P. Lin, M. Saimura, H. Ashida, T. Morii, *Bioorganic Med. Chem.* **2019**, *27*, 115120.

[17] Z. Zhao, J. Fu, S. Dhakal, A. Johnson-Buck, M. Liu, T. Zhang, N. W. Woodbury, Y. Liu, N. G. Walter, H. Yan, *Nat. Commun.* **2016**, *7*, 1–9.

[18] A. Ora, E. Järvihaavisto, H. Zhang, H. Auvinen, H. A. Santos, M. A. Kostiainen, V. Linko, *Chem. Commun.* **2016**, *52*, 14161–14164.
